# The effect of consuming evening primrose oil on cervical preparation before hysteroscopy: An RCT

**DOI:** 10.18502/ijrm.v20i7.11561

**Published:** 2022-08-08

**Authors:** Mandana Mansour Ghanaei, Maryam Asgharnia, Maryam Farokhfar, Seyed Mohammad Asgari Ghalebin, Elahe Rafiei, Katayoun Haryalchi

**Affiliations:** ^1^Reproductive Health Research Center, Department of Obstetrics and Gynecology, Alzahra Hospital, School of Medicine, Guilan University of Medical Sciences, Rasht, Iran.; ^2^Mehr Fertility Research Center, Guilan University of Medical Sciences, Rasht, Iran.; ^3^Razi Clinical Research Development Unit, Guilan University of Medical Science, Rasht, Iran.

**Keywords:** Hysteroscopy, Dilatation, EPOGAM, Gamma-linolenic acid.

## Abstract

**Background:**

Herbal medicine could be effective at treating various illnesses. Hysteroscopy can be an effective method for assessing the uterus in terms of anatomical, physiological and pathological anomalies.

**Objective:**

This study aims to evaluate the effect of evening primrose oil (EPO) on cervical preparation in women candidates for hysteroscopy.

**Materials and Methods:**

This study was a double-blind, randomized controlled clinical trial including 160 women candidates for diagnostic hysteroscopy who were referred to Alzahra hospital from August 2019-March 2020. They were divided into 2 groups. Group A received 100 mg EPO as a soft gel capsule 6 hr before the hysterectomy in the posterior vaginal fornix. Group B received a placebo. After receiving the treatment, primary and secondary outcomes were evaluated in the groups.

**Results:**

The average Hegar size in the EPO group was larger than in the control group (p < 0.001 for both). Also, the need for mechanical dilation, the time taken until the first resistance and the time of dilatation completion in the EPO group were significantly less than in the placebo group (p < 0.008 for all). There was also greater ease of dilatation in the EPO group. Side effects such as uterine rupture, false passage, cervical rupture, allergic reaction, abdominal pain, nausea, diarrhea, headache and increase of bowel movements were not reported in any cases.

**Conclusion:**

Based on the findings of the present study, EPO is effective for cervical preparation in women undergoing hysteroscopy.

## 1. Introduction

Hysteroscopy is a procedure in which a gynecologist evaluates the cervix by inserting a small telescope (hysteroscope) into the vagina and cervix (1). Approximately 50% of hysteroscopy complications are related to difficulties entering the cervix (2). Potential complications include cervical tears, false passage creation, uterine perforation, and difficulty in cervical entry (2-4).

Proper cervical ripening (CR) prior to hysteroscopy reduces the risk of complications associated with a difficult entry into the cervix (5). Cervical preparation is a complex process that results in physical softening, increased effacement, and dilatation due to enzymatic activity that decomposes collagen fibers and improves interstitial water (6, 7). CR agents include oral or vaginal prostaglandins that are either synthetic such as misoprostol, or natural like dinoprostone and vaginal osmotic dilators like laminaria (1, 8).

E series prostaglandins effectively dilate and prime the cervix, but some of them, such as dinoprostone, require special temperatures for preservation (9). In recent years, misoprostol (synthetic prostaglandin E1) has been used for obstetric and gynecological conditions such as in abortion therapy, treatment of postpartum hemorrhage as well as preparation treatment for hysteroscopy, vacuum aspiration, and dilatation (10-14). Some side effects of misoprostol include nausea, vomiting, diarrhea, vaginal bleeding, and abdominal cramps (11, 12). Misoprostol is the drug of choice for CR, but an alternative is needed due to its high price and instability (11, 12, 14). According to a meta-analysis, current evidence does not support routine administration of misoprostol before surgical hysteroscopy for all cases (15).

Evening primrose is a plant with yellow flowers that bloom at sunset. It is a native of North America and grows wild in parts of Europe (16). Iran has one of the most suitable climates for the cultivation of evening primrose (17).

Evening primrose oil (EPO) is a natural extract of the plant seeds. Because of its high content of polyunsaturated fatty acids, especially linoleic acid and gamma-linolenic acid as well as vitamin E, it is used as a dietary supplement (18). Gamma-linolenic acid is a well-known precursor to prostaglandin E (19) and is metabolized to arachidonic acid (20). The therapeutic effect of this plant is also attributed to the omega-6 essential fatty acid, which indirectly affects the synthesis of vaginal prostaglandins and cytokines (21). Consumption of EPO causes a significant increase in gammallenolic acid in the blood (22). A small number of contrasting studies have been published about the effect of EPO on CR during pregnancy (23-26). Some studies have also claimed that the use of EPO in CR prior to hysteroscopy is safe and effective (27-29).

It is important to find less expensive alternatives for prostaglandins with fewer side effects to use in CR to prepare for hysteroscopy. For this reason, and because of the shortage of studies into the effect of EPO on CR before hysteroscopy, it was decided to investigate the effect of administrating EPO on cervical preparation.

## 2. Materials and Methods 

This study was conducted using a double-blind, randomized controlled clinical trial design with 160 women who were referred to Alzahra hospital, Rasht, Iran and were candidates for diagnostic hysteroscopy from August 2019-March 2020.

Potential participants were assessed based on inclusion and exclusion criteria through clinical examinations and individual statements. The inclusion criterion was women candidates for hysteroscopy. The exclusion criteria were: present pregnancy, systemic diseases, cervical or vaginal infections, contraindications for the use of EPO (women with bleeding disorders, consumers of anticoagulants, schizophrenic individuals receiving phenothiazine, epileptics and those on medication for antihypertension), anomalies and cervical insufficiency, or Müllerian type of delivery in case of previous childbirth (28).

The randomization method used was the stratified blocked randomization method, using quadruple blocks for 160 cases. Eligible subjects were divided into 2 groups: EPO and placebo, based on random allocation. The website https://www.sealedenvelope.com was used to make a randomization list of persons divided into study groups.

The intervention group (A) received 1000 mg of EPO (provided by Barij Esans Pharmaceutical Company, Iran) in the form of a soft gel capsule (2 pieces of 500 mg each) 6 hr before hysteroscopy in the posterior fornix of the vagina. The control group (B) received a placebo (placebo capsule provided by Barij Esans Pharmaceutical Company, Iran) in the same dose and shape as the evening primrose capsules in the same way as the intervention group (28). The researchers and participants were not aware of the sample randomization.

The same surgeon performed the hysteroscopy under general anesthesia using a resectoscope with an 11 mm outer sheath and a 30 lens.

Cervical dilatation began by using a size 3 Hegar dilator. The number of the dilator at the first resistance and the interval between the first resistance and the dilatation generated with size 10 Hegar were also recorded.

Factors considered in the study included: the time until the first resistance, duration of dilatation until the use of a size 10 Hegar, duration of hysteroscopy, size of the first Hegar, ease of dilation, and the need for mechanical dilatation. These factors were regarded as the primary outcomes to be measured throughout the surgery. Complications in the uterus and cervix (uterine rupture or bleeding, cervical rupture, and false passage), drug-related side effects, and preoperative pain due to medication were considered as secondary outcomes. Secondary outcomes were measured during the surgery and throughout the 24 hr after the surgery. A surgeon then evaluated all outcomes.

### Ethical considerations

Ethical approval was obtained from the ethics committee of the Vice-Chancellor of Research at Guilan University of Medical Sciences, Rasht, Iran (Code: IR.GUMS.REC.1398.193). Informed consent was taken from all participants. All participants' information was kept anonymous.

### Statistical analysis

The data collected from the different stages were assessed, coded and analyzed using the SPSS software (Statistical Package for the Social Sciences, version 21.0, SPSS Inc, Chicago, Illinois, USA). The median and mid-quadratic ranges were used to describe the abnormally distributed quantitative variables. Qualitative variables were described based on number and percentage. The subgroups' normal distribution of the quantitative variables was measured using elongation and skewness, histogram diagrams, Q-Q plots, and the Wilk-Shapiro test. To compare the quantitative abnormal variables in both groups, the Mann-Whitney test, Chi-square test, and Fisher's exact test were used. The statistical significance of the tests was determined to be p < 0.05.

## 3. Results

**Figure 1:**
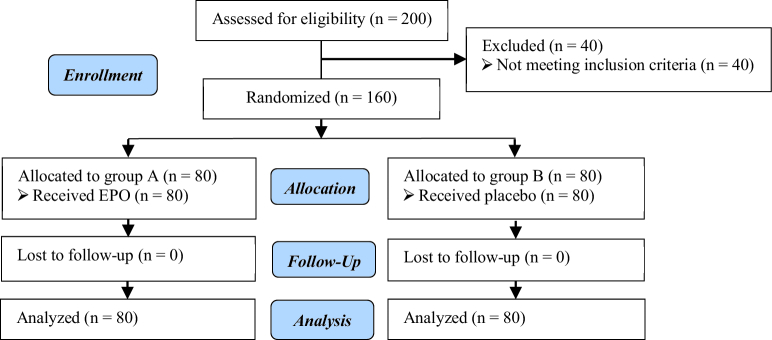
Diagram of the study population.

In this study, 200 subjects were interviewed. 40 did not meet the inclusion criteria, and 160 female candidates for hysteroscopy in 2 different groups of EPO and placebo completed the study. The selection process and status of the subjects in the study are given in figure 1.

In the female candidates for hysteroscopy there was no statistically significant difference in age (p = 0.57), body mass index (p = 0.58) and parity (p = 0.21) between the 2 groups receiving EPO and placebo. However, in terms of gravidity, there was a statistically significant difference between the 2 groups (p = 0.03) (Table I).

For the menopausal women who were candidates for hysteroscopy, there was a statistically significant difference between the 2 groups in gravidity, first Hegar size, first resistance enteral and dilatation interval. There was not a statistically significant difference between the 2 treatment groups in gravidity, regarded as a variable interfering in the linear relationship with factors under investigation in the study. In candidates for non-menopausal hysteroscopy, there was a significantly different first Hegar size, first resistance enteral and dilatation interval between the 2 groups (Table II).

The percentage of those requiring mechanical dilatation and the time of dilation in the EPO group were significantly lower than in the placebo group (p < 0.001) (Table III). Dilatation in the EPO group was generally easier than in the placebo group (p < 0.001) as 100% of the subjects did not report preoperative pain (n = 0). The difference in the level of preoperative pain in the EPO vs. control group was not statistically significant (p = 0.23). The percentage of adverse effects in the EPO group was higher than in the placebo group. Reported complications included 23 cases of spotting (15 cases in the EPO group and 8 cases in the placebo group) and 1 case of bleeding in the EPO group. However, the difference between the 2 groups was not statistically significant (p = 0.11). No side effects such as uterine rupture, cervical rupture, false passage, allergic reactions, abdominal pain, nausea, diarrhea, headache, or increased bowel movements were observed (Table III).

In the subgroup of menopausal candidates for hysteroscopy, there was no statistically significant difference between the 2 groups in terms of the size of the first Hegar used (p < 0.001) (Table III). The percentage of those needing mechanical dilatation in the EPO group was significantly lower than in the placebo group (p < 0.01). The time spent until the first resistance (p < 0.001) and the duration of completing the dilation (p < 0.001) in the EPO group were also significantly shorter than in the placebo one. The duration of the operation in the EPO group was shorter than in the placebo group; however, this difference was not statistically significant (p = 0.96).

There was greater ease of dilatation in the EPO group than in the placebo group (p < 0.001). The difference in the preoperative pain levels in the EPO and placebo groups was not statistically significant (p > 0.99). The percentage of side effects in the EPO group was higher than in the placebo group; however, the observed difference was not statistically significant (p = 0.47). Complications reported included 9 cases of spotting (6 cases in the EPO group and 3 cases in the placebo group). Side effects such as bleeding, uterine rupture, false passage, cervical rupture, allergic reactions, abdominal pain, nausea, diarrhea, headache, and increased bowel movements were not observed in any cases.

In the subgroup of non-menopausal women who were candidates for hysteroscopy, there was a statistically significant difference between the 2 groups in the size of the first Hegar used (p < 0.01). The percentage of the need for mechanical dilatation in the EPO group was significantly lower than in the placebo group (p = 0.01) (Table IV).

The time taken until the first resistance (p = 0.01), and the time of dilatation completion (p < 0.01) in the EPO group were also significantly shorter than in the placebo group. The duration of the operation in the EPO group was shorter than in the placebo one; however, the observed difference was not statistically significant (p = 0.13). There was a greater ease of dilatation in the EPO group than in the placebo group (p = 0.01). The preoperative pain in the EPO and placebo groups did not differ significantly (p = 0.19).

The percentage of side effects in the EPO group was higher than in the placebo one; however, the difference was not statistically significant (p = 0.24). Side effects included 13 cases of spotting (8 cases in the EPO group vs. 5 cases in the placebo group), and 1 case of bleeding was observed in the EPO group. Adverse effects such as uterine rupture, false passage, cervical rupture, allergic reactions, nausea, diarrhea, headache, and increased bowel movements were not reported.

**Table 1:** Clinical characteristics of the groups (n = 160)


**Variable**	**Group A (n = 80)**	**Group B (n = 80)**	**P-value**
**Age (yr)**	44 (38-56)	45 (37-55)	0.57
**BMI (kg/m^2^)**	28.00 (26.00-31.75)	27.50 (26.00-32.00)	0.58
**Gravidity**	2 (1-3)	1 (1-3)	0.03
**Parity**	1 (1-2)	1 (0-2)	0.21
Data presented as Mean (Interquartile range). Mann Whitney test, BMI: Body mass index			

**Table 2:** Clinical characteristics of the subgroups (n = 160)


	**Menopause (n = 60)**			**Non-menopause (n = 100)**		
**Variable**	**Group A** **(n = 30)**	**Group B** **(n = 30)**	**P-value**	**Group A** **(n = 50)**	**Group B** **(n = 50)**	**P-value**
	**Age (yr)**	57.5 (50.8-62.5)	57.5 (53.0-62.0)	0.87	38.5 (35.0-43.3)	37.5 (32.0-43.3)	0.33
**BMI (kg/m^2^)**	30.0 (26.8-36.0)	29.5 (26.0-38.0)	0.79	27.0 (26.0-29.3)	26.4 (25.8-30.0)	0.61
**Gravidity**	2.5 (2.0-4.0)	2.0 (1.0-3.0)	0.03	2.0 (1.0-2.3)	1.0 (0.3-2.0)	0.20
**Parity**	2.0 (1.0-2.0)	2.0 (1.0-2.3)	0.70	1.0 (0-2.0)	1.0 (0-2.0)	0.19
**First Hegar size**	2.0 (1.0-3.0)	1.0 (0-1.0)	< 0.01	2.0 (1.0-3.0)	1.5 (0.8-2.0)	< 0.01
**First resistance enteral (sec)**	20.0 (8.8-30.0)	49.0 (34.8-65.0)	< 0.01	12.5 (5.0-31.3)	20.0 (13.8-40.0)	0.01
**Dilatation interval (sec)**	30.0 (15.0-44.3)	70.0 (57.3-101.3)	< 0.01	25.0 (16.5-45.3)	40.0 (30.0-72.5)	< 0.01
**Operation duration (min)**	18.0 (15.0-23.0)	20.0 (15.0-22.3)	0.96	18.0 (15.0-20.0)	20.0 (15.0-20.0)	0.13
Data presented as Mean (Interquartile range). Mann Whitney test, BMI: Body mass index						

**Table 3:** Outcomes of the groups (n = 160)


**Variable**		**Group A (n = 80)**	**Group B (n = 80)**	*P-value
**Mechanical dilatation**		53 (66.3)	8 (10.0)	< 0.001
**Dilatation facility**				
	**Excellent**	21 (26.3)	3 (3.8)	
	**Good**	23 (28.7)	10 (12.5)	
	**Moderate**	20 (25.0)	24 (30.0)	
	**Week**	10 (12.5)	25 (31.3)	
	**Very weak**	6 (7.5)	18 (22.5)	< 0.001
**Side effects**		15 (18.8)	8 (10.0)		0.11
Data presented as n (%). *Chi-square test				

**Table 4:** Outcomes of the subgroups (n = 160)


		**Menopause (n = 60)**			**Non-menopause (n = 100)**		
**Variable**		**Group A (n = 30)**	**Group B (n = 30)**	*P-value	**Group A (n = 50)**	**Group B (n = 50)**	**P-value
**Mechanical dilatation**		23 (76.6)	30 (100)	0.01	30 (60)	42 (84)	0.008
**Dilatation facility**							
	**Excellent **	7 (23.3)	0	14 (28)	3 (6)	
	**Good**	8 (26.7)	0	15 (30)	10 (20)	
	**Moderate **	11 (36.8)	8 (26.8)	9 (18)	16 (32)	
	**Week **	3 (10.0)	13 (43.3)	7 (14)	12 (24)	
	**Very weak**	1 (3.3)	9 (30.0)	0.001	5 (10)	9 (18)	0.01
**Side effects**		6 (20)	3 (10)		0.47	9 (18)		5 (10)	0.24
Data presented as n (%). *Fisher's exact test, **Chi-square test							

## 4. Discussion

The increasing use of medicinal plants by women, especially during their reproductive periods and pregnancy, has attracted the attention of researchers in pursuit of more effective treatments (30). Modern medical sciences tries to treat individuals with the least invasive method possible.

The results showed a difference between the 2 groups in the size of the first Hegar used, which was larger in the EPO group. Also, the need for mechanical dilation, the time taken until the first resistance, and the time of dilatation completion in the EPO group were significantly less than in the placebo group. The dilatation in the EPO group was easier than in the placebo group. There was no significant difference between the 2 groups in terms of complications. There were similar results in the 2 groups in cases of menopausal candidates for hysteroscopy.

Most of the Iranian review studies conducted in recent years have focused on EPO with regards to premenstrual syndrome and polycystic ovary syndrome. The results of all those studies have confirmed the positive effect of EPO in reducing premenstrual syndrome symptoms (31, 32). Another review study conducted in 2019 indicated that EPO was generally effective in improving the health of Iranian women within 4-6 months at most (33). Few previous studies on EPO have concentrated on its effect on dilatation and cervical preparation before hysteroscopy (23). Therefore, more extensive studies are required in this regard.

A study on non-menopausal women without a history of normal vaginal delivery and on menopausal women under hysteroscopy confirmed the positive effect of EPO in preparing the cervix before hysteroscopy. Duration of cervical dilatation was shorter in the EPO group, and the size of the first Hegar used in that group was larger than in the placebo one (28). In the present study, the median length of Hegar was bigger in the EPO group.

A randomized clinical trial compared the performance of intracervical laminaria vs. intravaginal EPO before hysteroscopy. They showed that both medicines were effective in cervical dilatation. However, cervical dilatation was induced in a shorter time and more easily in the EPO group compared to in the laminaria group. The researchers also showed that EPO could even be used 6 hr before gynecological surgery procedures such as hysteroscopy and curettage to improve cervical dilation and firmness. About 85% of cases showed improvement in cervical dilatation and effacement as well as a change of the bishop score 4 hr after the intervention with the EPO capsule (34). In the present study, ease of dilatation was better in the EPO group.

Based on the results of the present study, there was no difference in the rate of complications between the 2 groups. Also, there were no side effects such as uterine rupture, false passage, cervical rupture, allergic reactions, abdominal pain, nausea, diarrhea, headache, and increased bowel movements. Similar results were reported in a pilot study on menopausal women and nulliparous women with menopause. In this study, no patient in the EPO group showed any adverse effects (29).

In the present study, the percentage of patients requiring mechanical dilation and the time taken to complete dilatation in the EPO group were significantly less than in the placebo one. In a double-blind clinical trial, there was similarly a statistically significant difference between the EPO and placebo groups (7.8 mm vs. 4.3 mm) due to the initial dilation of the cervix. In the EPO group, only 47.6% of subjects needed mechanical dilation of the cervix. The mean time to achieve a dilation of 10 mm was shorter in the EPO group (53.5 sec vs. 17.3 sec) (27). In another study, oral EPO and intracervical gel of dinoprostone were equally effective on cervical preparation before hysteroscopy (35).

One of the advantages of the present study was the use of EPO in a larger sample size compared to previous studies.

## 5. Conclusion

Based on the findings of the present study and other similar studies conducted in recent years, we can conclude that EPO is effective for cervical preparation in women candidates before hysteroscopy. Therefore, it is recommended as an alternative treatment to improve women's health because of its convenience and cost-effectiveness.

##  Conflict of Interest 

The authors declare that there is no conflict of interest.
